# On-farm testing of reduced animal welfare demands on productivity and welfare in pig production

**DOI:** 10.1186/s13028-025-00825-6

**Published:** 2025-08-02

**Authors:** Per Wallgren, Stefan Gunnarsson

**Affiliations:** 1 Swedish Veterinary Agency, SVA, 751 89 Uppsala, Sweden; 2 Department of Clinical Sciences, Swedish University of Agricultural Sciences (SLU), Box 7054, 750 07 Uppsala, Sweden; 3 Department of Applied Animal Science and Welfare, Swedish University of Agricultural Sciences (SLU), Box 234, 532 23 Skara, Sweden

**Keywords:** Animal density, Animal welfare, Confinement, Swedish legislation, Weaning age

## Abstract

**Background:**

Swedish animal welfare requirements exceed those of EU, which may have contributed to decreased pig production in Sweden since joining EU. On request from stakeholders, the Swedish Board of Agriculture allowed testing effects of reduced welfare demands on pig production for one year. This included weaning before 28 days at individual level, temporary confinements of sows during farrowing and mating, and increased stocking density of growers. The intervention period lasted for one year, and the productivity was compared with the preceding year.

**Results:**

A reduced mean weaning age from 32 to 27 days did not have a significant effect on piglet welfare measures but the annual number of piglets weaned per sow increased by 1.3. Temporary confinement of sows at farrowing had no significant effect on piglet mortality or productivity, but the confinement reduced sow welfare. Temporary confinement of sows during mating did not improve sow productivity. Instead, hygiene and welfare decreased due to the confinement. Decreased stocking density by 10% compared with Swedish requirements had no significant effect on welfare measures. Nor did weight gain differ from the previous year. However, increased batch size of fatteners increased the incidence of respiratory lesions at slaughter, impaired growth and feed conversion.

**Conclusions:**

Simulations assured that over 90% of the piglets were older than 25 days in batches weaned at a mean age of 28 days. As piglets mature from three to four weeks of age, this was important for piglet welfare. Short-term confinement of sows at farrowing or mating decreased sow welfare due to the confinement while no significant increase in piglet productivity was found. An increased stocking density by 10% of weaners and growers did not affect welfare measures but increased the number of potential disease transmissions between pigs by 22% due to increased batch sizes. When batch size increased, respiratory lesions at slaughter increased and productivity decreased. Based on the results, the Board of Agriculture allowed herds to wean at a mean age of 28 days provided they complied with special requirements. Except for this, the minimal legal requirements of pig welfare in Sweden remained unchanged.

## Background

Sweden has since 1988, higher animal welfare demands regarding minimum standards in pig production than most other countries [1, 2] and Sweden was also the first country that banned the use of growth promoters in animal feed already in 1986 [3–5]. Prior to 1995, the farm animal production in Sweden was subsidized, as well as protected, by duties on imported food, mainly motivated by national contingency plans for preparedness for warfare [6]. In 1995, Sweden joined the European Union, and consequently the Swedish farming was subjected to the Common Agricultural Policy (CAP) of the EU. However, the animal welfare legislation of Sweden from 1988 [1] was kept intact, and since then the pig production of the country decreased from 4.0 million pigs per year in 1994 to 2.6 million pigs in 2010 [7]. The decreased pig production is mainly believed to have been dependant on higher costs than in competing countries, *e.g*. lower productivity of sows due to the longer suckling period, increased space demands leading to increased building costs and higher labour costs. As the consumption of pork per capita was relatively stable, the proportion of imports of pig meat increased [7].

Because of the increasing competition from imported pork, the Swedish Pig Farmers Association (SPFA; Sveriges grisföretagare) by 2012 requested that the animal welfare regulations in Sweden should be adapted towards the minimum standards of the EU directive 2008/120 [8]. Differences in animal welfare demands comparing Sweden to the EU focused on the ban of crating farrowing sows in Sweden, as sows in Sweden always shall be loose housed although temporary confinement for *e.g.* veterinary treatment is allowed. Loose housing of sows during the major time of the gestation is a legal demand also within EU [8] but not implemented everywhere [9]. As a result of the differences in regulations, housing of sows demands larger spaces in Sweden, *i.e.* farrowing pens should not be smaller than 6 m^2^ [1] leading to increased building costs. Furthermore, the minimal age at piglet weaning is 28 days on individual level [1]. A minimal weaning age of 28 days corresponds to a farrowing rate of 2.3 times per sow and year, which is 11.5% lower compared to a mean weaning age of three weeks (2.6 litters per year) which is allowed within EU [2]. Additional demands of the Swedish animal welfare legislation include access to litter material and ban of tail docking [1].

**Fig. 1:**
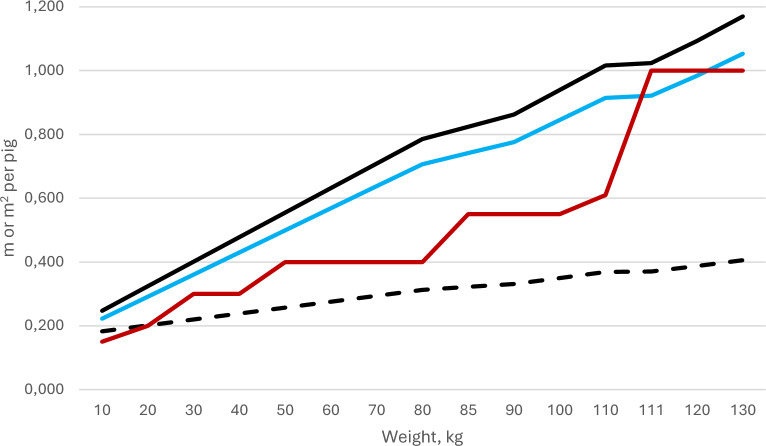
Minimal area requirements for growing pigs in Sweden and in EU. Minimal area requirements (m^2^) for growing pigs in Sweden (black line) and reduction of that with 10% (blue line). The red line shows the minimal area allowance for growing pigs within EU. The black dotted line shows the minimal through length (m) per pig in Sweden. No such minimal requirement is stated by the EC pig directive

The SPFA also demanded that the minimal space requirements for growers and fattening pigs should be decreased with the aim of reducing production costs. As seen in Fig. 1, also the area demands of weaned pigs and growers in Sweden exceeds the minimum demands within EU [1, 8] and also includes specified minimal length of the feed trough. No minimal requirement regarding minimal through length is stated by the EC pig directive.

With the intention to maintain a high biosecurity level, the animal welfare legislation of Sweden demands a maximum of 400 pigs per unit in specialized fattening enterprises that purchase growers at a weight of 25–30 kg body weight from more than one piglet producing herd [1]. New buildings are adapted to this demand, but the utility of old and larger buildings has been hampered, and therefore the SPFA wanted to abolish the restriction.

On request from SPFA, the Swedish Board of Agriculture (SBA) financed an on-farm intervention study [SBA dnr 5.2.18-3510/15] that aimed to evaluate the effects on both welfare and performance of reduced requirements than stated in the Swedish animal welfare legislation [1]. The study was effectuated by initiating a deviation from minimum requirements of the welfare legislation by allowing temporary crating of sows during farrowing and mating, decrease piglet age at weaning and increasing stocking density for growers and fatteners in specific commercial herds that were monitored during the study.

Thus, the aim of this manuscript was to describe and evaluate the recorded data about performance, animal health and welfare from the farms that applied the deviations of the minimal standards of the Swedish animal welfare legislation.

## Methods

### Design of intervention studies on farms

The study was financed by SBA [SBA dnr 5.2.18-3510/15 (2013–2341)] and carried out during the year 2014. It was effectuated by farmers appointed by the SPFA, and in cooperation with the animal health organisations Farm & Animal Health (Uppsala, Sweden) and Lunden Animal Health Ltd (Långås, Sweden). Results obtained were compiled by PIG (Pig Aligned Trials, Skara, Sweden). The results were evaluated by two independent validators (*i.e.* the authors of this document) from the Swedish Veterinary Agency (SVA) and the Swedish University of Agricultural sciences (SLU) that had access to all data obtained. Before the initiation of the study, the validators recommended that the performance records of the pig farms attained during the year that proceeded the study (2013) should be used for comparison within individual herds with the results obtained from the same herds during the intervention year (2014). The production records documented during 2013 were achieved under conditions complying with the minimal standard of the animal welfare legislation of Sweden [1], while the interventions were performed during 2014. Also, the number of potential disease transmission within batches was defined according to the formula n * (n-1) where n represented the number of pigs in the group [10].

### Farms in the intervention studies and registrations made

In total, eleven herds were included in the intervention study effectuated in 2014. No herd participated in all steps of the intervention studies. The steps of the intervention study that each herd participated in are shown in Table 1. All herds effectuated age segregated rearing, *i.e.* each batch of sows with suckling piglets, weaners and fatteners were housed in a previously emptied and cleaned unit separated from the other units of the site. During gestation, sows were housed with other sows in continuous systems. The size of the piglet producing and integrated herds ranged from 300 to 900 sows with an annual production of growers ranging from 8000 to 24,000 during the preceding year (2013). The size of the specialised fattening herd that purchased growers from piglet producing herds ranged from 1060 to 3000 pigs and the number of growers annually reared to market weight ranged from 3200 to 9000.

**Table 1:** Herds in the study and the intervention steps that they participated in

	Participation in the following intervention steps*					
Type of herd	1	2	3	4	5	6
1 Piglet producer	X	X		X		
2 Piglet producer		X		X		
3 Farrow to finish	X	X	X			
4 Farrow to finish		X		X	X	X
5 Piglet producer		X				
6 Piglet producer	X					
7 Piglet producer		X				
8 Piglet producer			X			
9 Piglet producer			X			
10 Finisher herd					X	X
11 Finisher herd					X	X

*The different steps of the study: (1) Earlier weaning; (2) Confinement of sows for up to five days at farrowing; (3) Confinement of sows for up to seven days at heat; 4) Space demands decreased with 10% for weaners; (5) Space demands decreased with 10% for fatteners; (6) allowance of purchase of more than 400 pigs per unit for specialised fattening enterprises

Each herd was visited by the Animal Health Organisation in charge at a monthly basis. During these visits, animal health and welfare measures included in the study (see below) were validated as no deviation (0) or as different degrees of deviation (1 = Minor deviation, 2 = Major deviation). The mean deviation score ± 1 standard deviation during the entire trial period were calculated for each parameter. To ensure a concordant estimation of the welfare measures, the animal health veterinarians were calibrated with each other before initiating the intervention study. As these recordings were initiated during the intervention year (2014), corresponding recording from the preceding year (2013) do not exist. However, none of the herds were attended for deviations from the welfare law during that year.

The production performance of the piglet production was registered as piglets born, weaned and reared to market weight per sow and year, weight at weaning and age at 30 kg body weight of piglets, as well as replacement incidence of sows and sows returning to oestrus. Weight at weaning and age at 30 kg body weight were defined by weighting six apparently normal litters per herd. The performance of growing pigs was registered as weight gain, feed conversion and mortality. The mean weight gain was achieved by multiplying the mean carcass weight with 1.34, withdraw the mean arrival weight and thereafter divide that figure with the number of rearing days for each batch. These production parameters were recorded by standard methods (PigVision®, AgroVision, Apeldoorn, The Netherlands) at each herd during both the intervention year (2014) and the preceding year (2013). In addition, the incidence of pathological lesions recorded at slaughter (pneumonia, pleuritis, abscesses, leg- and tail injuries) were documented by standards made by the Swedish Food Agency [11–13]

### Intervention 1: Decreased weaning age to below 28 days on individual level

According to the Swedish Animal Welfare Legislation, weaning must not take place before 28 days of age at an individual level [1]. In synchronized age segregated rearing systems, it takes about seven days from the farrowing of the first sow to the farrowing of the last sow in each batch. Therefore, a minimal age at weaning of 28 days on individual level leads to a mean age at weaning of 32 days. With a mean weaning at 32 days, sows farrow 2.3 times a year. Comparatively, sows weaned three weeks post farrowing, which is allowed within EU, will give birth to 2.6 litter per year. With the aim of improving sow productivity, weaning was allowed at a lower age than 28 days at an individual level, provided that the mean weaning age not was lower than 28 days. To provide the piglets with a satisfactory diet, the farmers were demanded to use a weaning feed with around 15% protein that included 4% lactose [14]. Simulations were made to estimate the incidence of piglets aged less than 28, 26 and 24 days, respectively, when applying weaning at a mean age of 28 days were estimated. These estimations were based on the spread of farrowing days from three consecutive farrowing batches (n = 40 sows per batch) when adjusted to a mean weaning age of 28 days in a herd with age segregated rearing, *i.e*. that weaned all sows in a group on the same day.

Three herds participated in this part of the study (Table 1). Animal welfare measures of sows were recorded in terms of hygiene, body condition score, side fat thickness, mortality and replacement of sows. In piglets, deviations in animal welfare measures were recorded in terms of weaning below 24 days of age (minor deviation) and below 22 days (major deviation), deviant behavior post weaning such as belly nosing or ear suckling and ambient temperatures at different sites of the unit. The production was recorded as described above (PigVision®) and compared with the performance during the preceding year.

### Intervention 2: Temporary crating of sows after farrowing for 5 days

In many countries, sows are often confined (crated or tethered) during farrowing and nursing with the aim of reducing building costs and piglet mortality. However, according to the Swedish Animal Welfare Legislation [1], sows must never be tied up or fixated in crates. To test if confinement of sows could reduce piglet mortality, sows were crated from the start of farrowing until a maximum of five days post farrowing during the trial. The allowed duration of the confinement was based on reports claiming that around 80% of the piglet mortality takes place during the first three days post-partum [15–19].

Six herds participated in this part of the study (Table 1). Of these, Herd 1 and 3 also applied a decreased weaning age as these herds also effectuated intervention 1. For that reason, these two herds were excluded from analyses regarding performance, but not regarding welfare. Animal welfare parameters were recorded in all herds as described above. To ensure comparison with the performance during the preceding year (2013), the performance was recorded by standard methods (PigVision®).

### Intervention 3: Confinement of sows during mating

According to the Swedish Animal Welfare Legislation, sows must never be confined [1]. During heat and under the influence of hormones, loose-housed sows are inclined to mount each other [20]. Low ranked sows/gilts therefore may be at risk of being injured. With the aim to reduce the incidence of injuries and replacement incidence of sows during heat, and thereby improve animal welfare, sows were confined in their feeding cubicles during the heat following weaning. Sows were confined when heat was suspected/detected in the first sow of the group.

Three herds participated in this part of the study (Table 1). All herds had loose house rearing of the sows, including an open area complemented with individual feeding cubicles sized 0.4 * 2.0 m (0.8 m^2^). Mounting was prevented by compiling the sows in the feeding cubicles for up to seven days during the heat post weaning. Parameters of animal health and welfare were recorded in terms of climate, hygiene, sow mortality and replacement of sows. The performance of the sows was recorded by standard methods (PigVision®) and compared with the performance during the preceding year (2013).

### Intervention 4: Increased stocking density during the post weaning period

The minimal area requirements for growing pigs according to the Swedish Animal Welfare legislation [1], are shown in Fig. 1. The genetic improvements of the previous decades had resulted in larger litters sizes [21] and if piglets of a litter should be kept intact, grower pens in buildings built when the expected litter’s sizes were smaller may not have legitimate space enough for all piglets born. The common solution at these farms has been to reduce the number of sows in farrowing batches with empty farrowing pens as consequence. With the aim to adapt the grower units to the increased reproductive performance and to house all piglets born within the same unit, the stocking density of weaners was allowed to increase by 10% during the post-weaning period in this study (see Fig. 1 that also shows the area minimum area demands for growing pigs within EU).

Three herds participated in this part of the study (Table 1). Animal health and welfare parameters were recorded in terms of density of pigs, behavior, mortality and tail biting. The performance was recorded by standard methods (PigVision®) to ensure comparison with the performance during the preceding year (2013).

### Intervention 5: Increased stocking density for fattening pigs

Similarly to the increased stocking density for weaners, the stocking density for fatteners was increased by 10% (see above).

Three herds participated in this part of the study (Table 1). Animal welfare issues and performance were recorded as described above.

### Intervention 6: Increased size of units for fattening pigs

As previously mentioned, specialized fattening herds that merchandise growers from more than one piglet producer are not allowed to have more than 400 growers per unit [1], which hampered the utility of larger buildings built before that ban. With the aim to improve the utility of such buildings, the limitation of 400 pigs per unit was abolished in herds with existing units larger than that.

Three herds participated in this part of the study (Table 1). Animal welfare parameters were recorded in terms of density of pigs, behavior, mortality and tail biting. The performance was recorded by standard methods (PigVision®) to ensure comparison with the performance during the preceding year (2013).

### Statistics

Summary statistics are presented as mean ± 1 standard deviation. Comparisons between control and trial groups were made by t-tests.

## Results

### Intervention 1: Decreased weaning age to below 28 days on individual level

**Fig. 2:**
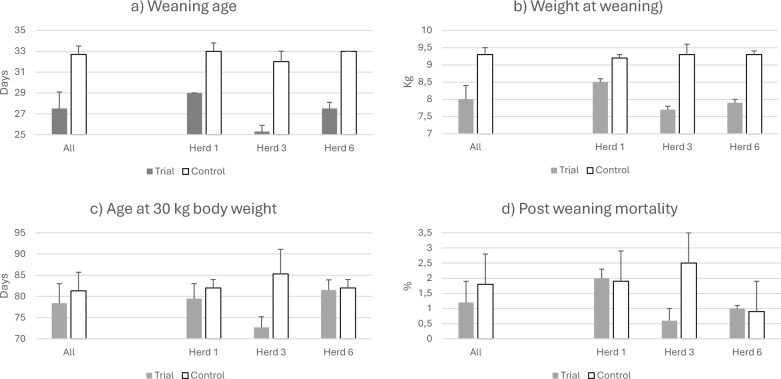
Decreased weaning age. Production parameters during the intervention year (2014) and the preceding year (2013)

The mean age at weaning decreased significantly (*p* < 0.001) from 32.7 ± 0.8 days during the preceding year to 27.5 ± 1.6 days during the intervention, corresponding to an increased annual farrowing rate from 2.3 to 2.4 per sow (Fig. 2a). At the observed mean number of piglets weaned per litter (11.6 during both the intervention year and the preceding year), this corresponded to an increased annual number of pigs weaned per sow and year from 26.5 ± 1.0 to 27.8 ± 1.1 per sow (*p* < 0.001). As the mean mortality during the post weaning period was 1.6%, this corresponded to a mean number of growers at a weight of 30 kg body weight per sow and year of 26.1 ± 1.0 (2013) and 27.4 ± 1.1 (2014), respectively (*p* < 0.001). Employing the mean mortality of 2% obtained during the fattening period in intervention 5, this corresponded to 25.6 ± 1.0 pigs reared to market weight per sow and year during the preceding year compared to 26.8 ± 1.1 during the intervention year (*p* < 0.001).

The weight at weaning was significantly lower during the intervention year due to the younger age at weaning (8.0 ± 0.4 kg vs 9.3 ± 0.2 kg; *p* < 0.001; Fig. 2b), but the age at 30 kg body weight did not differ significantly between the years (78.4 ± 4.6 vs 81.34.5 days; *p* = 0.15; Fig. 2c). Nor did the post weaning mortality differ significantly between the intervention and the preceding year (Fig. 2d) (Table 2).

**Table 2:** Deviations according to protocol in animal welfare parameters when applying an earlier weaning

	Deviations recorded		
Parameter	Herd 1	Herd 3	Herd 6
Weaning beneath 24 days	0	0.9 ± 0.4	0.4 ± 0.4
Non-slaughter of sows out of date*	0.3 ± 0.5	0	0
*Deviations in weaning pens*			
No disinfection between batches	0.2 ± 0.6	0	0
Not used to creep feed at weaning	0	0.2 ± 0.7	0
No lactose in creep feed	0	0	0
Temperature at lying area	0.5 ± 0.8	0	0.2 ± 0.7
Floor temperature	0	0.9 ± 0.8	0
Lying behavior post weaning	0	0.8 ± 1.0	0.4 ± 0.9
Belly nosing	0.2 ± 0.4	0	0.2 ± 0.4
Ear suckling	0.1 ± 0.3	0	0

Deviation score: 0 = No deviation; 1 = Minor deviation; 2 = Major deviation

*None slaughter of sows selected for slaughter, *i.e.* because of non-pregnancy of other sows

The simulations regarding expected incidence of litters aged less than 28 days at weaning is shown in Table 3. In mean 2.5% of the litters were aged less than 26 days when weaned. The mean incidence of litters aged less than 24 days was 1.7% and was caused by two gilts (*i.e.* first parity sows) that farrowed late in the third batch.

**Table 3:** Simulated individual weaning ages when applying weaning at a mean age of 28 days

Age of litters at weaning	Batch I	Batch II	Batch III	Merged
Aged < 28 days at weaning	19 of 40 (47.5%)	15 of 40 (37.5%)	6 of 40 (15%)	40 of 120 33.3%
Aged < 26 days at weaning	1 of 40 (2.5%)	0 of 40 (0%)	2 of 40 (5%)	3 of 120 2.5%
Aged < 24 days at weaning	0 of 40 (0%)	0 of 40 (0%)	2 of 40 (5%)	2 of 120 (1.7%)

The simulations were calculated from true variations in weaning days in three consecutive batches of farrowing sows in a herd with age segregated rearing

Sows lost 0.22 ± 0.15 mm of their side fat thickness during the suckling period, both during the intervention and during the preceding year. Regarding piglet welfare, there were minor deviations (mean deviation values < 1 on herd level) regarding age at weaning (21–23 days, but not below 21 days) in two of the three herds. Postweaning, minor deviations were recorded with respect to floor temperature and temperature at the lying area for the piglets, as well as piglet behavior such as belly nosing and ear nosing, but none of these deviations were determined in all herds. There were also minor deviations regarding habituation to creep feed, but again not in all herds (Table 2).

### Intervention 2: Temporary crating of sows after farrowing for 5 days

**Fig. 3:**
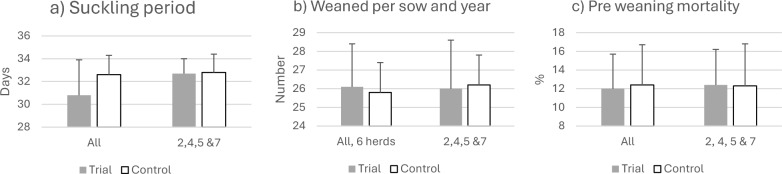
Production parameters when confining sows for a maximum of five days at farrowing. The two bars to the left represent all six herds that effectuated confinement. As Hard 1 and 3 also applied a shorter suckling period, they are excluded in the two bars to the right in each figure

The number of live born piglets were equal during the two years compared; 13.2 ± 0.8 during the intervention year and 13.3 ± 0.8 during the preceding year. Overall, the mean length of the suckling period decreased from 32.2 ± 1.8 days during the preceding year to 30.3 ± 3.6 days during the intervention (Fig. 3a), with a numerically higher number of piglets weaned per sow (Fig. 3b). However, it must be considered that herd 1 and herd 3 also employed a shortened suckling period. When these herds were excluded from the production analyses, the annual number of piglets produced per sow was numerically higher when not confining sows (Fig. 3b). There were no significant differences in number of piglets weaned per sow and year (26.2 ± 1.6 vs 26.0 ± 2.6, Fig. 3b) or in pre-weaning mortality (Fig. 3c).

As seen in Table 4, no major deviations were observed regarding the animal welfare parameters measured, but the overall welfare of the sows was reduced due to the confinement at farrowing.

**Table 4:** Deviations according to protocol in animal welfare parameters when confining sows at farrowing

	Deviations recorded (with exception of confinement)					
Parameter	Herd 1	Herd 2	Herd 3	Herd 4	Herd 5	Herd 7
Improper confinement of sows	0	0.2 ± 0.6	0	0.2 ± 0.6	0	0.3 ± 0.8
Straw at last twice daily	0	0	0	0	0	0
Water flow to sows (L/ min)	0.6 ± 0.9	0.6 ± 0.7	0	0.6 ± 0.8	0	0.4 ± 0.5
Caretaking routines of piglets	0	0	0	0	0	0
Temperature in the unit	0	0.3 ± 0.5	0.1 ± 0.3	0	0	0
Temp, lying area of piglets	0.6 ± 0.8	0.4 ± 0.8	0.8 ± 0.8	0.4 ± 0.7	0.8 ± 0.7	0.1 ± 0.4
Lying behaviour of piglets	0	0	0	0.5 ± 1.0	0	0
Stress related symptoms	0	0	0	0	0	0

Sows were confined for a maximum of five days at farrowing. Deviation score: 0 = No deviation; 1 = Minor deviation; 2 = Major deviation

### Intervention 3: Confinement of sows during mating

The production parameters of the sows did not significantly differ between the preceding and intervention years: 83% pregnancy, 6% returners to heat, 2% mortality and 9% taken out of production for other reasons; 13.5 live born piglets per litter whereof 11 were weaned. As seen in Table 5, no or minor deviations were recorded for most of the welfare measures recorded. However, continuous major deviations regarding cleanliness of sows were recorded during the trial year, supported by a reduced ability to manipulate straw or straw equivalents. These deviations contributed to terminating this part of the project during the summer of 2014.

**Table 5:** Deviations according to protocol in animal welfare parameters when confining sows at heat

	Deviations recorded* (with exception of confinement)		
Parameter	Herd 3	Herd 8	Herd 9
Confinement for longer than 7 days	0	0	0
Poor equipment standard	0	0	0
Wanted water flow in nipples (L/ min)	0	0	0
Condensation of air humidity in the unit	0	0	0.1 ± 0.3
Ability for rooting / manipulating straw	0.3 ± 0.7	0	0–2 ± 0.6
Wounds on sows	0	0	0
Hygiene, cleanliness of sows	**1.6 ± 0.9**	**1.4 ± 1.0**	**1.6 ± 0.8**
Stress related behaviour of sows	0	0.1 ± 0.4	0.2 ± 0.4
Sow behaviour during heat	0	0	0.5 ± 0.8

*Deviation score: 0 = No deviation; 1 = Minor deviation; 2 = Major deviation

### Intervention 4: Increased stocking density during the post weaning period

**Fig. 4:**
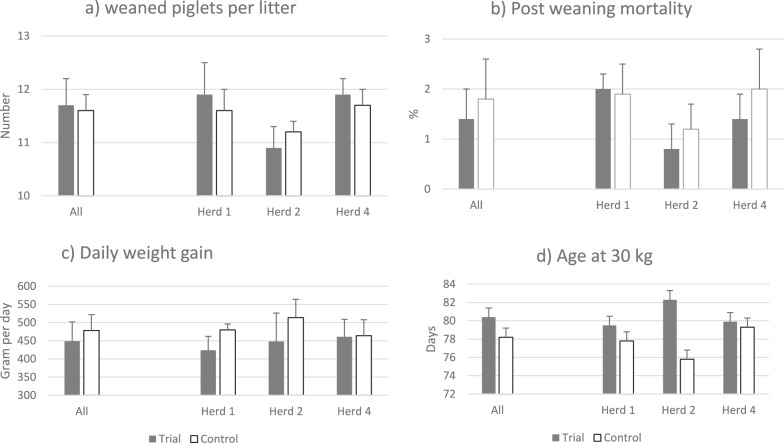
Production parameters when density of weaners was increased with 10% compared with the preceding year

The intervention and preceding years did not differ significantly in number of piglets weaned per litter (Fig. 4a) or post-weaning mortality (Fig. 4b). In contrast, the mean DWG post weaning was numerically (478 ± 44 vs 449 ± 53 g per day; *p* = 0.09) higher during the preceding year (Fig. 4c), resulting in a non-significantly increased mean age at 30 kg body weight with two days during the intervention year (78.2 ± 3.6 vs 80.4 ± 6.7 days; *p* = 0.25; Fig. 4d).

As seen in Table 6 there were minor deviations due to presence of tail biting in all herds during the intervention (mean deviation < 0.25 at herd level). No other deviations regarding the animal welfare parameters were recorded.

**Table 6:** Deviations regarding animal welfare associated qualities when pig density of weaners increased by 10%

	Deviations recorded		
Parameter	Herd 1	Herd 2	Herd 4
Calculated n of pigs per m^2^	0	0	0
True density of pigs per m^2^	0	0	0
Deviation from EU regulations	0	0	0
Water flow in nipples (L/ min)	0	0	0
Ability for rooting (access to straw)	0	0	0
Ability for all pigs to eat simultaneously	0	0	0
Tail biting lesions	0.1 ± 0.3	0.1 ± 0.3	0.2 ± 0.4

Deviation score: 0 = No deviation; 1 = Minor deviation; 2 = Major deviation

### Intervention 5: Increased stocking density for fattening pigs

**Fig. 5:**
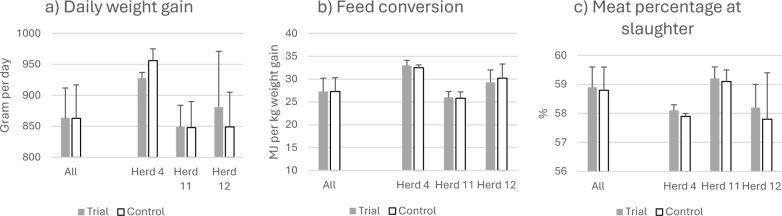
Increased stocking density of fatteners. Production parameters during intervention and the preceding year

There were no significant differences in performance between the intervention and the preceding years (Mean overall DWG = 864 ± 48 vs 862 ± 54 g per day, both groups consumed 27.3 MJ metabolizable energy per kg growth and market weight pigs had a meat percentage of 59% (Fig. 5). Nor did the incidence of lesions registered at slaughter differed significantly between the years.

As seen in Table 7, there were minor deviations regarding tail biting, water flow in nipples and one remark regarding a density of pigs above the Swedish regulation plus 10% — but still lower than the EU legislation.

**Table 7:** Deviations regarding animal welfare associated qualities when pig density of fatteners increased by 10%

	Deviations recorded		
Parameter	Herd 4	Herd 10	Herd 11
Calculation of pigs per m^2^	0	0	0
True density of pigs per m^2^	0	0.1 ± 0.6	0
Deviation from EU regulations	0	0	0
Water flow in nipples (L/ min)	0.4 ± 0.7	0.6 ± 0.7	1.2 ± 1.0
Ability for rooting (access to straw)	0	0	0
Ability for all pigs to eat simultaneously	0	0	0
Tail biting lesions	0.8 ± 0.4	0.4 ± 0.4	0.5 ± 0.5

Deviation score: 0 = No deviation; 1 = Minor deviation; 2 = Major deviation

### Intervention 6: Increased size of units for fattening pigs

Complete data was obtained from Herd 4, whereas Herd 10 and 11 were discarded due to missing data. During the intervention, the integrated Herd 4 allocated piglets born to other herds and instead purchased growers to one isolated building for fatteners with two units. Six batches with piglets produced at the herd 2013 were compared with six batches of merchandised fatteners during intervention (2014). An equal number of growers entered the units (n = 480) which corresponded to 230,000 potential transmissions between pigs, compared to 160,000 potential transmissions in batches with 400 pigs.

No welfare deviations were recorded during the intervention year, but the growth performance was numerically reduced when compared to the preceding year (DWG of 959 ± 45 vs. 982 ± 48 g per day; *p* = 0.10) and the feed conversion ratio was impaired (26.5 ± 2.8 vs. 24.2 ± 2.1 MJ per kg feed per kg weight gain; *p* < 0.05). The carcass weight was numerically lower during the intervention (89.3 ± 4.0 vs. 91.5 ± 2.5 kg; *p* = 0.10). In contrast, the meat percentage of carcass bodies was higher during the intervention (58.7 ± 0.7 vs. 57.9 ± 0.5%; *p* < 0.05).

The mortality during rearing was numerically higher during the intervention year (1.6 ± 0.8% vs. 1.0 ± 0.8%; *p* = 0.09) and the incidence of pigs condemned at slaughter was higher during the intervention (0.2 ± 0.1% vs. 0.1 ± 0.1%; *p* < 0.05). At slaughter, the incidence of pigs with remarks of pneumonia, with mycoplasma-like lesions increased from 2.3 ± 1.6% during preceding year to 8.3 ± 5.8% during intervention (*p* < 0.01), and the incidence of lesions resembling acute Actinobacillosis increased from 0.1 ± 0.2% to 1.1 ± 2.2% (*p* < 0.05). The incidence of tail injuries registered at slaughter, presumably mainly caused by tail biting, was numerically higher during the intervention (3.0 ± 2.3% vs. 2.1 ± 1.2%; *p* = 0.22).

## Discussion

Although the Swedish report of the evaluation has been available in public via SBA since 2015, the results that emanate from different organisations have in practice been kept within them since then. Now, ten years after the effectuation of the intervention project, the authors have received permission to publish the comparison between the intervention with the preceding year from all stakeholders. Ideally, the study ought to have been larger and longer (more herds and more years) to ensure more thorough detection of challenges in the interactions between health, welfare and productivity. Productivity has of course improved since 2014, *e.g.* regarding piglets born per litter and daily weight gain. Nevertheless, we consider that the results obtained are still valuable as they were obtained on commercial farms at conditions that were applicable to practical pig farming in Sweden, and records and experiences were gained in a way that no experimental setup would be able to achieve. The results obtained can be considered valuable as no similar on-farm study previously has been made in Sweden.

Testing weaning at an earlier age than the legally required age was motivated by larger litters and increased weight of piglets at weaning achieved by breeding, resulting in higher strain on the sows in terms of more milk required by the litter during lactation [22]. When applying a weaning age of at least 28 days and age segregated rearing, the true weaning age will range from 28 to 35 days with synchronised weaning of farrowing batches. When the mean weaning age decreased from 32 to 27 days, no improved welfare of sows could be determined in terms of a reduced loss of side fat thickness of sows during the lactation. This result was positive since it indicated that the farmers managed to feed the sows appropriately during lactation. The risk for PWD and deviations in terms of belly nosing and/or ear suckling in piglets increase with lower weaning age [23] but may occur also in piglets weaned at 28 days of age [23–26]. From the aspect of the piglets, the important issues include access to nourishment and a proper environment. A younger age at weaning resulted in lighter piglets at weaning, but the age when reaching 30 kg body weight did not differ between the groups. Overall, no significant deviations regarding animal welfare were recorded, and the piglet mortality pre and post weaning did not differ between intervention and the preceding year. However, the younger age at weaning increased the annual number of piglets weaned per sow by approximately 1.3 piglets, making it tempting to recommend the allowance of weaning at a younger age than 28 days on an individual level.

Still, piglets are immature when born and a validation made by EFSA from 2007 [27] concluded that piglets ought not to be weaned before 28 days of age. Concordantly the directive of EU demands that piglets should not be weaned before 28 days of age for welfare reasons unless the wellbeing of sow or piglets is judged as jeopardized [8]. Nevertheless, the EU directive opens for weaning at 21 days of age if piglets are transferred to empty and cleaned facilities. Still, the immatureness of the piglets must be considered, and piglets aged four weeks are considerably more mature than piglets aged three weeks [23], as also reflected by a lower mortality from weaning and onwards and a higher daily weight gain during the fattening period in Sweden than in countries applying weaning at three weeks of age based on statistics from Eurostat [28].

The Swedish legislation of weaning at a minimum age of 28 days was established in 1988 when sows usually farrowed individually and not in age segregated groups. This weaning age ought, according to the results obtained, be possible to reduce somewhat without jeopardizing animal welfare. However, the difference in maturity between three and four weeks of age [23] ought to be considered. Not the least since it was also reflected in Herd 3 and 6 of this study with deviations for weaning at a younger age than 24 days concurrently with a higher incidence of unwanted behavior of weaners than in Herd 1 without deviations for weaning piglets younger than 24 days. Therefore, the authors recommended allowance of a change from weaning at 28 days on an individual level to 28 days at batch level if the creep feed includes lactose. A transformation like that will improve productivity without seriously jeopardizing welfare as the simulations made proved that over 95% of the piglets will be 26 days or older at weaning.

Sows have commonly been confined with the aim to decrease pre-weaning mortality of piglets. However, as around 80% of the piglet mortality take place during the first three days of life [15–19] confinement of sows was limited to a maximum of five days. The minimum size of farrowing pens in Sweden is 6.0 m^2^, and during those conditions confining sows for a maximum of five days at farrowing did not decrease piglet morality. That result concurred earlier reports concluding that confining sows in farrowing pens sized > 6 m^2^ not affected pre-weaning mortality [29–32]. Consequently, confining sows at farrowing appeared to be un-called for, and piglet mortality ought rather to be combatted by introducing larger farrowing pens in countries with farrowing crates sized < 5 m^2^ or preferably even < 6 m^2^. The continuous enlargement of sow body sizes and litter sizes rather indicate a desire of increased sizes of farrowing pens, not only for piglet welfare reasons but also with the aim to improve productivity. A redesign of farrowing pens from 6.85 m^2^ to 8 m^2^ in a Swedish herd had no impact on the pre-weaning mortality, but the weaning weight at a mean age of 31 days was 10.4 kg (weight gain of 287 per day) compared to 8,9 kg before introducing the enlarged pens (weight gain of 239 g per day[36]. As a comparison, the corresponding national mean was 8.2 kg at the age of 32.8 days (weight gain of 204 g per day) [33].

In systems with groups of loose-housed sows, high ranked sows tend to attack low ranked sows during eating to steal their fodder. Therefore, sows are confined in individual feeding cubicles during eating. As loose-housed sows also tend to mount each other during heat [20], confining sows during heat could be one way to protect low ranked sows and thereby increase animal welfare during this period. In the present study, sows were confined in their feeding cubicles during heat. However, apart from reduced possibilities to manipulate straw/straw equivalents, the hygiene went out of control and that part of the study was therefore terminated before the end of intervention period. If sows are to be confined during heat, the cubicles obviously need to be improved (larger and with improved possibilities to remove faeces) compared with the standard of the feeding cubicles aimed for confinement temporarily during eating in Sweden of today.

As litter sizes increase, existing buildings designed for a lower productivity may not have space enough to harbour the offspring according to legal demands with full occupancy of sows. One way to handle this is to decrease the size of the farrowing batches of sows, with decreased herd sizes as consequence. As the minimal area demands for growing pigs are higher in Sweden compared to the minimum standard of the EU directive (Fig. 1), a decreased area per pig with 10% were tested for weaners and fatteners. When doing so, the area demands were still higher than the EU directive (Fig. 1). For both age categories, there were minor deviations regarding tail biting, but the incidence of tail injuries recorded at slaughter were not higher than the mean of 3% of the country [34]. The deviations regarding water flow in nipples were not desirable and demonstrated the need for controlling water flow to properly provide pigs with water, but that deviation was not related to animal density. For these reasons, we concluded that an increased pig density with 10% in existing buildings that had increased their production above the levels expected when built could be acceptable from an animal welfare perspective, provided that all pigs could eat simultaneously*, i.e.* the legal demands on space allowance to feeding trough length were still to be maintained (Fig. 1). However, we also concluded that such an exception from the regulations would increase the number of potential transmissions between pigs with 22% in each unit, and as productivity increase over time; no exceptions regarding space were recommended at construction of new buildings.

Regarding specialised fattening herds purchasing growers from several piglet producers, the legislation [1] demands a maximum of 400 pigs per unit for biosecurity reasons which has hampered the utility of large old buildings in specialised fattening herds. A unit size exceeding400 fatteners was analysed in an integrated herd with units of 480 growers that during the intervention allocated weaners and instead merchandised growers to these units. The number of potential transmissions between pigs in these units were 44% higher than in units with 400 pigs (230,000 vs. 160,000). There were no severe differences regarding welfare issues detected during the intervention, but the incidence of respiratory lesions recorded at slaughter was increased and the feed conversion was impaired. The DWG during the intervention period was decreased with 23 g (2.3%), which corresponded to a prolonged rearing period of two days compared with the preceding year. Taken together, these observations indicated a possible higher risk for production errors of increased batch sizes when merchandising pigs of potentially unknown origin. One could of course argue that the carcass meat percent was higher during the intervention year, but that rather indicated a negative impact of the increased pathogen load mirrored by an increased incidence of respiratory diseases registered at slaughter, a lower DWG and an inferior feed conversion compared to the preceding year. Thus, the results obtained did not indicate allowance of buildings with units larger than 400 pigs. From a visionary perspective, instead bisecting existing units with 400 pigs into two units with 200 pigs per unit appear tempting as it will reduce the number of potential transmissions between pigs with 75% compared to unit sizes of 400 (from 160,000 to 40,000). Indeed, the incidence of pathologic lesions registered at slaughter turned very low in a farrow to finish herd that at rebuilding established fattening units with mechanical ventilation and only 40 pigs per unit which reduced the number of transmissions with 99% compared to units with 400 stalls (1600 vs 160,000) [27, 28].

Following the report, SBA decided not to allow any deviations from the animal welfare legislation [1] regarding confinement of sows or increased density of growing pigs, motivated by the fact that such measures decreased welfare due to confinement of sows and decreased individual space for growers without improving productivity. Consequently, no herd effectuate confinement of sows by today. Concurrently, no herd practise an increased density of weaners or fatteners. Interestingly, some integrated herds have instead decreased the density among their fatteners with around 10% by allocation of surplus growers to specialised fattening herds. In accordance with the ending of the paragraph above, these herds are reported to have experienced increased weight gain and tranquillity among pigs, (Gunnar Johansson, Farm & Animal Health, personal communication). The increased tranquillity was evidently beneficial when rearing pigs with intact tails as in Sweden. Tail docking is prohibited within EU since 2008, but as tail docking is allowed when judged to be required [8], the ban is only effectuated in a few countries like Finland and Sweden [35, 36]. However, as tail docking do not eliminate tail biting [37, 38], and as the incidence of tail biting in Sweden at a national level does not exceed that of other EU countries [35], there is a growing support for allowing pigs to keep their tails worldwide — primarily for animal welfare reasons [39, 40].

Following the report, SBA decided that one exception from the animal welfare legislation [1] could be implemented in herds that were inspected by an animal health organisation at least every third month controlling the welfare status of the herds. This exception included weaning piglets at a minimal age of 21 days, however with a maximum of 10% younger than 26 days – *i.e*. corresponding to a mean age of 28 days at weaning. Now, ten years after, 19 herds practise weaning at a mean age of 28 days (Helena Elofsson, SBA, personal communication). This number would probably have been higher in absence of the demands made by SBA. However, these requirements are meant to ensure compliance to the intention of the animal welfare legislation.

## Conclusions

The results of this study indicated that a mean weaning age of 28 days in farrowing batches did not have any negative effect on piglet welfare when the creep (weaning) feed included lactose. This earlier weaning improved the productivity of the sows and is today implemented in 19 herds that document their production and are controlled quarterly by an animal health organisation.

Confining of sows at farrowing decreased sow welfare without increasing productivity, as no effect on piglet mortality was found. Nor was confining sows during mating, as applied in this trial, supported for hygienic reasons. Because of the study results, the authorities kept the legislation prohibiting confinements of sows intact.

The results indicated that an increased density of weaners and fatteners with 10% (0.9 m^2^ at 100 kg) compared with legal issues of Sweden (1.0 m^2^ at 100 kg) was acceptable from a welfare perspective but also increased the number of transmissions between pigs with 22%, indicating a risk for increased spread of infections if introduced and thereby a risk for higher pathogen load. Indeed, the incidence of respiratory diseases registered at slaughter was higher and the DWG and feed conversion was lower in large fattening units (485 pigs per unit) when recruited by purchase of growers from the open market during the intervention than during the preceding year when the units were recruited from the own herd (one source). For these reasons, the authorities kept the area demands of the welfare legislation. Thus, these parts of the program are not implemented in any herd by today. Instead, some herds have decreased the pig density to above the legal demands experiencing increased weight gain and tranquility among pigs.

## Data Availability

The datasets used and analyses are available from the corresponding author on reasonable request.

## Abbreviations

CAP: Common agricultural policy
DWG: Daily weight gain
MJ: Megajoule
PWD: Post weaning diarrhoea
SBA: Swedish Board of Agriculture
SLU: Sveriges Lantbruksuniversitet (Swedish University of Agricultural Sciences)
SPFA: Swedish pig farmers association
SVA: Swedish Veterinary Agency
WOAH: World Organisation for Animal Health
